# Anxiety, Distress, and Turnover Intention of Healthcare Workers in Peru by Their Distance to the Epicenter during the COVID-19 Crisis

**DOI:** 10.4269/ajtmh.20-0800

**Published:** 2020-08-18

**Authors:** Jaime A. Yáñez, Asghar Afshar Jahanshahi, Aldo Alvarez-Risco, Jizhen Li, Stephen X. Zhang

**Affiliations:** 1 Carrera de Educacion y Gestion del Aprendizaje, Facultad de Educacion, Universidad Peruana de Ciencias Aplicadas, Lima, Peru;; 2 Teoma Global, Gerencia Corporativa de Asuntos Científicos y Regulatorios, Lima, Peru;; 3 CENTRUM Católica Graduate Business School (CCGBS), Pontificia Universidad Católica Del Peru (PUCP), Lima, Peru;; 4 Carrera de Negocios Internacionales, Facultad de Ciencias Empresariales y Economicas, Universidad de Lima, Lima, Peru;; 5 Tsinghua University, Beijing, China;; 6 University of Adelaide, Adelaide, Australia

## Abstract

We conducted a cross-sectional survey to assess the anxiety, distress, and turnover intention (likelihood to leave their current job) of healthcare workers in Peru during the COVID-19 pandemic. Our results reported that 21.7% healthcare workers in Peru experienced severe anxiety, whereas 26.1% of them experienced severe mental distress. A higher level of education related with a lower level of anxiety. Younger workers had a higher level of turnover intention than their older colleagues did. Healthcare workers in the private sector had a higher turnover intention than those in the public sector. Most importantly, people who were geographically far from Lima, the epicenter in Peru, during the outbreak experienced less anxiety and mental distress, corroborating the ripple effect and disconfirming the typhoon eye theory. However, the direction of these relationships can change depending on the type of institutions (public versus private) and the type of employees’ contract (full time versus part time). Our research helps provide insights for clinical professionals in identifying the vulnerable groups to mental disorders in Peru. This is the first study to assess anxiety, mental distress, and turnover intention in healthcare workers in Peru during the COVID-19 pandemic.

## INTRODUCTION

Peru has enabled the most restrictive measures in its national public health history to control the current COVID-19 outbreak.^1–4^ The first patient with COVID-19 in Peru was detected in Lima on March 5, 2020.^5^ Five days after, classes in schools were suspended nationwide, and on March 12, all classes at universities were suspended nationally.^6^ On March 15, a state of emergency, border closure, and lockdown was declared with the order of social isolation for 15 days,^7^ which has been extended multiple times, and currently, it has been announced to be until June 30, 2020. Those measures were very similar to the ones imposed in China, which have affected people’s lives, jobs, health, and well-being,^8^ increasing stress and anxiety^9,10^ during the COVID-19 outbreak.

Because of the social isolation in Peru, the COVID-19 crisis is expected to affect people’s mental health, especially that of healthcare workers. After the first case was detected on March 5, 2020, the confirmed cases increased rapidly overwhelming healthcare workers. Furthermore, personal protection equipment (PPE) access, burnout due to long work hours, not seeing their families for many days, the high risk of becoming infected, and the psychological harm of uncertainty have been reported to affect the physical and psychological status of healthcare workers in China^11^ and Iran.^11,12^ However, at the best of our knowledge, this has not been properly studied in Peru, as it has already been reported in China,^13–15^ Singapore,^16^ Iran,^12,17^ Italy,^18,19^ France,^20^ United Kingdom,^21^ and Spain.^22,23^ The COVID-19 crisis is causing an increase in burnout or anxiety,^24,25^ which resulted in an unprecedented psychological impact,^26–28^ and affecting people’s life satisfaction that is one of the most critical indicators of mental health.^29,30^ We aim to use early evidence in Peru to help mental health service provide in screening people with psychological issues during the COVID-19 outbreak from a novel perspective of typhoon eye theory.^13,31–33^ It has been observed that people who reside far from the epicenter of an outbreak usually overestimate the likelihood of becoming infected,^34^ which has been reported for COVID-19^13^ and earthquakes.^32^

This study identifies the vulnerable regions where individuals are more likely to suffer from well-being issues and helps to guide medical professionals’ attention toward the more mentally vulnerable groups based on the distance from the epicenter in the COVID-19 outbreak in Peru: Lima (the capital of Peru). The “ripple effect” refers to the phenomenon that the mental health issues are more problematic for people around the center of the epicenter, which was the case for mental health services during the SARS and Ebola outbreaks.^35–37^ However, because tremendous amount of social media exposure and information have been perceived during the COVID-19 outbreak,^38^ our research group already has reported that in China individuals’ well-being deteriorates over distance from the epicenter,^13^ as depicted by the psychological typhoon eye theory.^31,32^ In this study, we test whether the typhoon eye theory works and for whom it works in the COVID-19 outbreak in Peru.

We performed our analysis on healthcare workers as they are a COVID-19 vulnerable group. We selected age as a variable because it has been reported that the younger population are usually more adaptive to a natural disaster or to the outbreak of a virus.^39,40^ However, younger population also tend to access information on COVID-19 more frequently via digital sources such as social media,^41^ which causes them to be exposed to more negative content.^42^ Family size is an indicator of social support that one could receive during crisis like the current one^43^ because it served as an important resource to buffer stress and anxiety.^44^ We surveyed healthcare workers in 15 of the 24 provinces in Peru; these locations vary in their travel distance from the epicenter of Lima (0–1,292 km). We used anxiety, distress, and turnover intention scales to assess the mental health of healthcare workers in Peru after 1 month of lockdown and social distancing measures. Turnover intention is defined as the likelihood of an employee to leave his current job.^45^ Overall, drawing from psychological typhoon eye,^31,32^ this study provided a snapshot of adult healthcare workers’ mental health during the ongoing COVID-19 pandemic to enable more targeted mental health support in Peru.

## METHODS

### Study design.

We conducted a cross-sectional survey from April 10, 2020 to May 2, 2020, after 1 month of lockdown and social distancing measures, in Peru because of the COVID-19 outbreak. At the beginning of the survey (April 10), the number of confirmed cases in Peru was 5,897 and 169 deaths,^46^ whereas at the end of the survey (May 2), the number of cases increased to 42,534 and the number of deaths increased to 1,200.^47^ We surveyed healthcare workers from 15 Peruvian provinces with varying severities of COVID-19. Lima, the capital of Peru, was considered the epicenter of the COVID-19 outbreak because the first case of COVID-19 in Peru appeared in Lima. It also continuously reported the highest number of COVID-19 patients during the survey, with 4,210 cases (71.4% of total cases) on April 10^46^ and with 26,908 cases (63.3% of total cases) on May 2.^47^

### Participants.

The online survey reached 400 healthcare workers in healthcare organizations such as hospitals, clinics, first emergency responders, medical wards, nursing home, dental clinics, pharmacies, and other healthcare institutions. We received responses from 303 of them (response rate of 75%), who worked in 111 healthcare facilities, including 55 healthcare facilities in Lima, 33 healthcare facilities in Loreto, and 23 healthcare facilities from the other 22 cities (at least one from each city). Their distance to the epicenter (Lima) ranged from 0 km to 1,292 km. All survey participants provided their informed consent before the enrollment. The survey was approved by the Tsinghua University Ethics Committee (#20200322). The participants remained anonymous and had the option to finish the survey at any time, and their information was kept confidential. The participants were not involved in any of the planning, execution, and reporting stages of the study.

### Outcomes and covariates.

Healthcare workers’ anxiety, distress, and turnover intention were assessed using the seven-item Generalized Anxiety Disorder (GAD-7) scale,^48^ the Kessler Psychological Distress scale (K6),^49^ and the two-item turnover intention scale,^50^ respectively. The total score of anxiety was considered as normal (0–4), mild (5–9), moderate (10–14), and severe (15–21), whereas the psychological distress was considered as low (< 5), moderate (5–12), and serious (≥ 13). The cutoff value to consider the presence of anxiety was 10^51^ and 13 for psychological distress.^52^

The healthcare workers reported their age, gender, family status, education, occupation, type of healthcare organization (public or private), job level (entry, junior, intermediate, senior, and chief), exercise hours per day in the past week, and chronic health issues (yes or no). Education included the categories of high school, technical, bachelors, medical specialty, masters, and doctorate. Participants reported whether they had any chronic disease because comorbidities increase the chance of complications in a person with COVID-19^53^ and because people with ongoing medical issues could be more anxious. Using their work locations, we calculated the distance of their cities to the epicenter of Lima for each participant.

### Statistical analysis.

Data analysis was performed in STATA version 16.0 (StataCorp LLC, College Station, TX) with a significance level set at *P* < 0.05, and all tests were two-tailed. We used linear regression to predict anxiety, distress, and turnover intention using unweighted data.

## RESULTS

Table 1 presents the descriptive characteristics of the participating healthcare workers. Of the 303 healthcare workers, 53 (17%) were physicians, 63 (21%) were nurses, 63 (21%) were pharmacists, 80 (26%) were technicians, 20 (7%) were volunteers, and 24 (8%) were others. Most participants were female (194 [64%]), aged 25–44 years (229 [76%]), single without any children (107 [35%]) or married with more than one child (75 [25%]), had bachelor degrees or higher (235 [78%]), and worked full time (210 [69%]) in public healthcare institutions (213 [70%]). The average distance of the participants to the epicenter of Lima was 424 km, with a SD of 490 km. The participants scored an average of 15.4 (SD of 4.6) in the GAD-7 anxiety scale, and the average surpassed the cutoff of severe anxiety at 15.^51^ In the K6 distress scale, the participants scored an average of 19.2 (SD of 4.5), higher than the cutoff of mental distress disorder at 13.^52^

**Table 1:** Characteristics of responders

Characteristic	Count, *n* (%)									
	Total	Occupation						Location		
		Physician	Nurse	Pharmacist	Technician	Volunteer	Others*	Lima	Loreto	Other
Overall	303 (100)	53 (17)	63 (21)	63 (21)	80 (26)	20 (7)	24 (8)	158 (52)	110 (36)	35 (12)
Gender										
Men	109 (36)	28 (26)	19 (17)	18 (17)	23 (21)	9 (8)	12 (11)	44 (40)	49 (45)	16 (15)
Women	194 (64)	25 (13)	44 (23)	45 (23)	57 (29)	11 (6)	12 (6)	114 (59)	61 (31)	19 (10)
Age-group (years)										
18–24	9 (3)	0 (0)	1 (2)	1 (2)	2 (3)	2 (10)	3 (13)	1 (1)	6 (5)	2 (6)
25–34	93 (31)	11 (21)	13 (21)	14 (22)	40 (50)	7 (35)	8 (33)	51 (32)	32 (29)	10 (29)
35–44	136 (45)	25 (47)	41 (65)	27 (43)	22 (28)	10 (50)	11 (46)	77 (49)	47 (43)	12 (34)
45–54	52 (17)	13 (25)	6 (10)	18 (29)	14 (18)	1 (5)	0 (0)	23 (15)	20 (18)	9 (26)
> 55	13 (4)	4 (8)	2 (3)	3 (5)	2 (3)	0 (0)	2 (8)	6 (4)	5 (5)	2 (6)
Family status										
Single without any children	107 (35)	10 (19)	21 (33)	17 (27)	35 (44)	13 (65)	11 (46)	72 (46)	22 (20)	13 (37)
Single with one child	24 (8)	4 (8)	2 (3)	3 (5)	7 (9)	6 (30)	2 (8)	6 (4)	18 (16)	0 (0)
Single with more than one children	4 (1)	3 (6)	0 (0)	1 (2)	0 (0)	0 (0)	0 (0)	2 (1)	0 (0)	2 (6)
Married without any children	6 (2)	3 (6)	0 (0)	1 (2)	1 (1)	0 (0)	1 (4)	4 (3)	1 (1)	1 (3)
Married with one child	37 (12)	7 (13)	9 (14)	12 (19)	7 (9)	0 (0)	2 (8)	24 (15)	9 (8)	4 (11)
Married with more than one children	75 (25)	16 (30)	20 (32)	18 (29)	18 (23)	0 (0)	3 (13)	38 (24)	28 (25)	9 (26)
Divorced or others	50 (17)	10 (19)	11 (17)	11 (17)	12 (15)	1 (5)	5 (21)	12 (8)	32 (29)	6 (17)
Number of children										
0	119 (39)	15 (28)	21 (33)	22 (35)	36 (45)	13 (65)	12 (50)	81 (51)	23 (21)	15 (43)
1	80 (26)	15 (28)	16 (25)	18 (29)	16 (20)	7 (35)	8 (33)	34 (22)	40 (36)	6 (17)
2	59 (19)	11 (21)	11 (17)	21 (33)	13 (16)	0 (0)	3 (13)	28 (18)	22 (20)	9 (26)
3	31 (10)	6 (11)	10 (16)	2 (3)	12 (15)	0 (0)	1 (4)	8 (5)	18 (16)	5 (14)
4	10 (3)	4 (8)	4 (6)	0 (0)	2 (3)	0 (0)	0 (0)	6 (4)	4 (4)	0 (0)
> 4	4 (1)	2 (4)	1 (2)	0 (0)	1 (1)	0 (0)	0 (0)	1 (1)	3 (3)	0 (0)
Education level										
High school	1 (0)	0 (0)	0 (0)	0 (0)	1 (1)	0 (0)	0 (0)	0 (0)	0 (1)	0 (0)
Technical	67 (22)	2 (4)	3 (5)	1 (2)	41 (64)	1 (5)	9 (38)	25 (16)	40 (36)	2 (6)
Bachelors	148 (49)	16 (30)	42 (67)	32 (51)	28 (35)	19 (95)	11 (46)	87 (55)	47 (43)	14 (40)
Specialty	33 (11)	24 (45)	5 (8)	3 (5)	0 (0)	0 (0)	1 (4)	19 (12)	10 (9)	4 (11)
Masters	52 (17)	11 (21)	13 (21)	25 (40)	0 (0)	0 (0)	3 (13)	27 (17)	12 (11)	13 (37)
Doctorate	2 (1)	0 (0)	0 (0)	2 (3)	0 (0)	0 (0)	0 (0)	0 (0)	0 (0)	2 (6)
Work level										
New	37 (12)	5 (9)	0 (0)	4 (6)	4 (5)	20 (100)	4 (17)	11 (7)	25 (23)	37 (12)
Junior	26 (9)	3 (6)	1 (2)	4 (6)	15 (19)	0 (0)	3 (13)	20 (13)	3 (3)	26 (9)
Intermediate	190 (63)	30 (57)	58 (92)	29 (46)	61 (76)	0 (0)	12 (50)	101 (64)	70 (64)	190 (63)
Senior	21 (7)	5 (9)	1 (2)	12 (19)	0 (0)	0 (0)	3 (13)	15 (9)	3 (3)	21 (7)
Chief	29 (10)	10 (19)	3 (5)	14 (22)	0 (0)	0 (0)	2 (8)	11 (7)	9 (8)	29 (10)
Type of contract										
Part time	93 (31)	10 (19)	18 (29)	12 (19)	50 (63)	0 (0)	3 (13)	54 (34)	35 (32)	4 (11)
Full time	210 (69)	42 (81)	45 (71)	51 (81)	30 (38)	20 (100)	21 (88)	104 (66)	75 (68)	31 (89)
Type of institution										
Public	213 (70)	37 (59)	50 (79)	50 (79)	40 (50)	19 (95)	18 (75)	74 (47)	107 (97)	32 (91)
Private	90 (30)	26 (41)	13 (21)	13 (21)	40 (50)	1 (5)	6 (25)	84 (53)	3 (3)	3 (9)
City										
Lima	158 (52)	23 (43)	38 (60)	38 (60)	47 (59)	0 (0)	12 (50)	NA	NA	NA
Loreto	110 (36)	23 (43)	19 (30)	6 (10)	31 (39)	20 (100)	11 (46)	NA	NA	NA
Other	35 (12)	7 (13)	6 (10)	19 (30)	2 (3)	0 (0)	1 (4)	NA	NA	NA
Distance to the epicenter (km)										
0	165 (54)	24 (45)	41 (65)	41 (65)	47 (59)	0 (0)	12 (50)	158 (100)	0 (0)	7 (20)
1–300	7 (2)	2 (4)	0 (0)	4 (6)	1 (1)	0 (0)	0 (0)	0 (0)	0 (0)	7 (20)
301–600	7 (2)	2 (4)	0 (0)	4 (6)	1 (1)	0 (0)	0 (0)	0 (0)	0 (0)	7 (20)
601–900	7 (2)	1 (2)	3 (5)	2 (3)	0 (0)	0 (0)	1 (4)	0 (0)	0 (0)	7 (20)
901–1,200	116 (38)	24 (45)	19 (30)	11 (17)	31 (39)	20 (100)	11 (46)	0 (0)	110 (100)	6 (17)
> 1,200	1 (0)	0 (0)	0 (0)	1 (2)	0 (0)	0 (0)	0 (0)	0 (0)	0 (0)	1 (3)

NA = not applicable.

* Includes other occupations such as medical technologists, dentists, psychologists, biologists, administrators, ambulance drivers, physician auditors, students, and providers of general services.

### Predictors of anxiety, distress, and turnover intention.

The regression results in Table 2 examined the predictors of anxiety, mental distress, and turnover intention of healthcare workers in Peru during the COVID-19 outbreak. Education level had a negative association with anxiety (β = −0.746, CI: −1.441 to −0.050, *P* = 0.036). The effects of gender, age, work level, type of contract, and type of institution on anxiety were not significant. In the case of mental distress, the predictors (gender, age, education level, work level, type of contract, or type of institution) were not significant. There was a negative association between age and turnover (β = −0.033, CI: −0.057 to −0.008, *P* = 0.009), with younger healthcare workers (β = 2.817, CI: 2.452 to 3.181) aged among 18–24 years in comparison to 35–44 years (β = 2.162, CI: 1.946 to 2.378). Healthcare workers in the private sector had a higher turnover intention than those in the public sector (β = −0.420, CI: −0.810 to −0.031, *P* = 0.035). The effects of gender, education level, and work level on turnover intention were not significant.

**Table 2:** OLS regression results on the GAD-7 scale, K6 scale, and turnover intention of health workers by gender, age, number of children, education level, work level, type of contract, type of institution, and distance to the epicenter

Variable	Count, *n* (%)	Parameter estimates (95% CI)		
		GAD-7 scale	K6 scale	Turnover intention
Gender	303 (100)	0.734 (−0.472 to 1.939)	0.350 (−1.119 to 1.819)	−0.098 (−0.431 to 0.235)
Age-group (years)	303 (100)	−0.008 (−0.097 to 0.081)	−0.011 (−0.119 to 0.097)	−0.033* (−0.057 to −0.008)
18–24	9 (3)	6.989 (5.670 to 8.307)	10.157 (8.551 to 11.764)	2.817 (2.452 to 3.1819)
25–34	93 (31)	6.906 (6.284 to 7.528)	10.045 (9.287 to 10.803)	2.489 (2.317 to 2.661)
35–44	136 (45)	6.824 (6.043 to 7.605)	9.933 (8.981 to 10.885)	2.162 (1.946 to 2.378)
45–54	52 (17)	6.742 (5.190 to 8.294)	9.821 (7.929 to 11.712)	0.834 (1.406 to 2.263)
> 55	13 (4)	6.660 (4.255 to 9.064)	9.709 (6.778 to 12.639)	1.507 (0.843 to 2.171)
Number of children	303 (100)	0.035 (−0.540 to 0.611)	−0.061 (−0.763 to 0.640)	0.069 (−0.090 to 0.228)
Education level	303 (100)	−0.746† (−1.441 to −0.050)	−0.322 (−1.170 to 0.526)	0.008 (−0.184 to 0.201)
Technician	67 (22)	7.894 (6.805 to 8.984)	10.444 (9.117 to 11.772)	2.360 (2.060 to 2.661)
Bachelors	148 (49)	7.149 (6.557 to 7.740)	10.122 (9.401 to 10.843)	2.369 (2.205 to 2.532)
Masters	52 (17)	6.403 (5.709 to 7.097)	9.800 (8.955 to 10.646)	2.377 (2.186 to 2.569)
Doctorate	2 (1)	5.657 (4.400 to 6.914)	9.478 (7.947 to 11.010)	2.386 (2.038 to 2.733)
Work level	303 (100)	0.305 (−0.348 to 0.959)	0.458 (−0.339 to 1.254)	−0.109 (−0.289 to 0.072)
Type of contract	303 (100)	0.230 (−1.202 to 1.662)	−0.354 (−2.099 to 1.391)	−0.078 (−0.473 to 0.318)
Part time	93 (31)	−0.003‡ (−0.005 to −0.001)	−0.004§ (−0.007 to −0.002)	0.000 (−0.000 to 0.001)
Full time	210 (69)	−0.001 (−0.004 to 0.002)	0.000 (−0.004 to 0.004)	0.000 (−0.001 to 0.001)
Type of institution	303 (100)	0.633 (−0.778 to 2.044)	0.412 (−1.307 to 2.131)	−0.420^‖^ (−0.810 to −0.031)
Public	213 (70)	0.004 (−0.001 to 0.010)	0.008 (0.001 to 0.015)	−0.003^¶^ (−0.005 to −0.002)
Private	90 (30)	−0.000 (−0.002 to 0.001)	0.000 (−0.002 to 0.002)	−0.001^¶^ (−0.001 to −0.000)
Distance to the epicenter (km)	303 (100)	0.004 (−0.002 to 0.010)	0.007 (−0.000 to 0.014)	−0.003^¶^ (−0.005 to −0.002)
0	165 (54)	7.070 (6.440 to 7.701)	10.176 (9.407 to 10.945)	2.508 (2.334 to 2.682)
1–300	7 (2)	6.393 (5.671 to 7.115)	9.304 (8.424 to 10.184)	2.567 (2.368 to 2.767)
301–600	7 (2)	5.716 (4.532 to 6.900)	8.432 (7.000 to 9.874)	2.626 (2.299 to 2.953)
601–900	7 (2)	5.039 (3.296 to 6.781)	7.560 (5.437 to 9.683)	2.685 (2.204 to 3.166)
> 901	117 (39)	4.362 (2.032 to 6.691)	6.688 (3.850 to 9.527)	2.744 (2.101 to 3.388)
Interaction effects of type of institution and type of contract				
Distance to the epicenter × type of institution	303 (100)	−0.005** (−0.010 to −0.000)	−0.008†† (−0.014 to −0.002)	0.002‡‡ (0.001 to 0.004)
Distance to the epicenter × type of contract	303 (100)	0.002 (−0.001 to 0.005)	0.004§§ (0.000 to 0.008)	0.000 (−0.001 to 0.001)

GAD-7 = generalized anxiety disorder; K6= psychological distress; OLS = ordinary least squares.

* *P* = 0.009.

† *P* = 0.036.

‡ *P* = 0.011.

§ *P* = 0.002.

‖ *P* = 0.035.

¶ *P* = 0.000.

** *P* = 0.049.

†† *P* = 0.01.

‡‡ *P* = 0.001.

§§ *P* = 0.029.

### The distance to the epicenter as a predictor.

First, margin analysis revealed the relationship between the distance to the epicenter and anxiety was significantly negative, a ripple effect, taking all the other covariates equal (β = −0.002, CI: −0.004 to −0.0002, *P* = 0.031). This relationship, however, might vary when other variables changed. The regression results in Table 2 indicated a significant interaction effect between the distance to the epicenter and the type of institution on anxiety (β = −0.005, CI: −0.010 to −0.000, *P* = 0.049). The interaction effect between the distance to the epicenter and job contract (full time versus part time) on anxiety was not significant (β = 0.002, CI: −0.001 to 0.005, *P* = 0.223). Yet, margin analysis showed the relationship between the distance to the epicenter and anxiety was significant and showed a ripple effect only among full-time healthcare workers (β = −0.003, CI: −0.005 to −0.001, *P* = 0.011) and not among temporary healthcare workers (β = −0.001, CI: −0.004 to 0.002, *P* = 0.583).

Second, margin analysis revealed the relationship between the distance to the epicenter and anxiety was significantly negative, a ripple effect, taking all the other covariates equal (β = −0.003, CI: −0.005 to −0.004, *P* = 0.023). This relationship, also, varied when other variables changed. The regression results in Table 2 indicated a significant interaction effect between the distance to the epicenter and the type of institution on anxiety (β = −0.008, CI: −0.014 to −0.002, *P* = 0.010). Margin analysis showed the relationship between the distance to the epicenter and anxiety was significant and showed a typhoon eye effect only among healthcare workers in public institutions (β = 0.008, CI: 0.001 to 0.015, *P* = 0.021) and not among healthcare workers in private institutions (β = 0.000, CI: −0.002 to 0.002, *P* = 0.883). The interaction effect between the distance to the epicenter and job contract (full time versus part time) on anxiety was also significant (β = 0.004, CI: 0.000 to 0.008, *P* = 0.029). Moreover, margin analysis showed the relationship between the distance to the epicenter and anxiety was significant and showed a ripple effect only among full-time healthcare workers (β = −0.004, CI: −0.007 to −0.002, *P* = 0.002) and not among temporary healthcare workers (β = 0.001, CI: −0.004 to 0.004, *P* = 0.955).

Third, margin analysis revealed the relationship between the distance to the epicenter and turnover was not significant taking all the other covariates equal (β = 0.002, CI: 0.0003 to 0.001, *P* = 0.494). The regression results in Table 2 indicated a significant interaction effect between the distance to the epicenter and the type of institution on turnover (β = 0.002, CI: 0.001 to 0.004, *P* = 0.001). Margin analysis showed the relationship between the distance to the epicenter and turnover was significantly negative among healthcare workers in both public institutions (β = −0.003, CI: −0.005 to −0.002, *P* = 0.000) and private institutions (β = −0.001, CI: −0.001 to −0.000, *P* = 0.000).

## DISCUSSION

Since 2012, Peru implemented the community mental health model as recommended by the WHO^54^ as an approach to provide care in the community through specialized facilities called community mental health centers. However, as reported in 2019, actual full-time employees of these centers reported that there are critical barriers that still need to circumvent.^55^ Some of them include lack of consistent training, resources, structure, and policies that effectively support the use and importance of these centers in the evaluation and adequate treatment of mental health conditions.^55^

This situation also gets worsened when general practitioners in Peru consider themselves as not very competent in diagnosing and treating mental disorders. This was reported in a self-perception survey that evaluated the competence of Peruvian general practitioners in diagnosing and treating major depression, anxiety disorder, alcohol dependence, and schizophrenia.^56^ It is reported that of the 434 responders, 70.5% believed they were competent in diagnosing depression, 73.3% for anxiety, 67.6% for alcohol dependence, 62.0% for schizophrenia, and when the four mental disorders were combined, only 41.6% of participants self-perceived competence in providing an adequate diagnosis.^56^ These results highlighted the need to improve medical education so as to develop the skills necessary to confront mental health disorders.^56^ There is a very limited number of studies that have assessed mental health in the general public and healthcare workers in Peru, and, to the best of our knowledge, this study is the first to report the mental health of healthcare workers in Peru during the COVID-19 outbreak.

Our study shows that overall people who were geographically further from the epicenter in Peru during the outbreak experienced less anxiety and mental distress, corroborating the ripple effect and disconfirming the typhoon eye theory.^11,13,31–33^ However, this relationship can change depending on the type of institutions (public versus private) and contract (full time versus part time). The relationship between the distance to the epicenter and distress for healthcare workers in public institutions was positive, showing a typhoon eye effect (β = 0.008, CI: 0.001 to 0.015, *P* = 0.021). Distance to the epicenter is a crucial factor for psychiatrists to consider to screen the mentally vulnerable groups,^13,30,57^ but research needs to first establish whether the distance to the epicenter carries a ripple effect or a typhoon eye effect. Furthermore, our results indicate that healthcare workers with a lower education level were more anxious, and younger healthcare workers and those in the private sector were more susceptible to turnover.

An important factor to consider is that at the beginning of the survey (April 10), the number of confirmed cases in Peru was 5,897 with 169 deaths,^46^ whereas at the end of the survey (May 2), the number of cases increased to 42,534 and the number of deaths increased to 1,200.^47^ This significant increase in confirmed cases and the accompanying coverage in the national and international media could have also caused increased anxiety and distress in healthcare workers. In addition, the reported precarious health system and saturation of every hospital in Peru with COVID-19 patients^3^ could have also caused an increase in turnover intention for healthcare workers. Similar to Iran,^17^ China,^13^ and the United States,^58^ we did not identify a universal risk factor that could predict specific mental disorders in Peruvian healthcare workers. This is expected as each country has their own medical system, clinical capacity, access to PPE, labor conditions, lockdown policies, and cultures.

### Limitations.

The context of this study has a clear epicenter of COVID-19, Lima, in Peru. However, it is not always the case as observed in South Korea.^59^ Our data were collected in Peru, a geographically large country, and it remains unclear whether the typhoon eye effect or the ripple effect will generalize in other countries, most of which are smaller. The epicenter of Lima is in the midwest of Peru, whereas the epicenter of Wuhan is in the middle of China, and the epicenter of New York State in the United States is in the northeast. Thus, we suspect that either the typhoon eye effect or the ripple effect might play out differently in term of pace and patterns.

## CONCLUSION

Our results show that Peru’s healthcare workers’ anxiety and mental distress decreased as the distance from the epicenter increases, corroborating the ripple effect and disconfirming the typhoon eye theory. A lower education level increased the anxiety levels, whereas age and gender did not affect the anxiety and distress levels. The turnover intention was not associated with the distance to the epicenter nor gender, but it was higher in younger healthcare workers in the private section. Our results can help guide mental health service providers toward vulnerable groups of healthcare workers that are closer to Lima, the COVID-19 epicenter in Peru. We urge for more research to assess the mental health of healthcare workers and general public in Peru, a country that was not given the importance it deserves.
